# Monte Carlo validation of signal-to-noise improvements in transmission imaging using correlated annihilation photon pairs

**DOI:** 10.1038/s41598-026-49361-x

**Published:** 2026-04-29

**Authors:** Gregory Romanchek, Zhicong Yu, Shiva Abbaszadeh

**Affiliations:** 1 Department of Nuclear, Plasma, and Radiological Engineering, University of Illinois Urbana-Champaign, Urbana, IL 61801 USA; 2 Department of Electrical and Computer Engineering, University of California Santa Cruz, Santa Cruz, CA 95064 USA

**Keywords:** Mathematics and computing, Physics

## Abstract

Quantum-Assisted Photon Imaging (QAPI) leverages the correlated photon pairs produced by positron annihilation to overcome the intrinsic noise limitations of classical radiation imaging. In this study, we develop a statistical framework describing the photon-counting behavior of QAPI and compare its predicted signal-to-noise ratio (SNR) performance against classical imaging under both idealized and realistic detector conditions. Analytical derivations demonstrate that QAPI exhibits reduced variance through two mechanisms: elimination of stochastic uncertainty in photon generation via idler detector measurements, and application of binomial rather than Poisson transmission statistics enabled by the high transmission probability of 511 keV photons. To validate these predictions, we performed GATE Monte Carlo simulations using a phantom with variable-depth inserts across a range of exposure times. Under idealized conditions, measured SNRs closely matched theoretical expectations, with QAPI consistently outperforming classical imaging across all transmission probabilities. Minor deviations at extreme transmission values were attributed to finite sampling effects and breakdown of the Poisson approximation. Realistic simulations incorporating CZT detector response revealed additional challenges, particularly coincidence pairing failures due to detector transmission, which we addressed through geometric correction of missing idler events and sensitivity-based normalization. Despite these complications, QAPI retained a substantial SNR advantage approaching $2\times$ improvement over classical imaging. These results establish that the statistical advantage of QAPI arises fundamentally from access to idler information and the favorable transmission characteristics of high-energy photons, providing a validated theoretical and computational foundation for quantum-assisted transmission imaging and motivating further experimental development.

## Introduction

One of the fundamental challenges in X-ray imaging is reliable detection and delineation of tissues or lesions when their attenuation properties are similar to those of the surrounding background. X-ray imaging traditionally relies on material-dependent photon attenuation, where varying absorption and scattering alter the transmitted radiation intensity^1^. Detectors capture these variations to construct an image. However, when differences in X-ray attenuation coefficients are small, the resulting contrast is minimal and subtle anatomical features become difficult to distinguish^2,3^. This problem is exemplified by the “white-on-white” appearance of small cancers in dense breast tissue, where limited transmission contrast obscures diagnostically critical features. The drive to minimize radiation dose without compromising diagnostic efficacy makes this challenge even more pronounced.

Conventional dose reduction strategies have substantially advanced the field, including: hardware advancements, image processing algorithms, and noise reduction techniques. The transition from analog to digital radiography utilizing flat-panel detectors has improved dose efficiency and image quality through higher detective quantum efficiency^4,5^. Advanced image processing methods such as noise reduction algorithms and dual-energy subtraction have further enabled dose reduction^6–9^, and more recently, artificial intelligence and deep learning-based post-processing have shown promise in reducing noise and enhancing contrast at lower radiation exposures^10–12^. Despite these advances, conventional X-ray imaging remains fundamentally constrained by quantum noise and scatter^13^. Quantum noise arises from statistical variation in the number of detected photons and constitutes the dominant noise source in X-ray imaging. Scatter, resulting from photons deviating from their original path after interacting with tissue, further degrades image quality and persists despite anti-scatter grids and post-processing corrections. These challenges represent classical limitations inherent to conventional imaging. However, recent advancements in quantum-enhanced imaging techniques show promise in overcoming this intrinsic noise limitation^14,15^.

Quantum correlation of photons, particularly in entangled photon pairs, offers a powerful tool for enhancing imaging capabilities beyond classical limits. Quantum correlation refers to the phenomenon where the properties of two or more quantum particles become linked such that the state of one particle provides information about the state of the other(s). In quantum imaging, correlated photon pairs exhibit strong interdependence in properties such as polarization, momentum, and frequency^16^. Among techniques exploiting quantum correlation is ghost imaging^15,17,18^, so named because photons that have not interacted with the object contribute to image formation^14^. In ghost imaging, photons interacting with the object (*signal photons*) are detected by one detector, while correlated photons that have not interacted with the object (*idler photons*) are recorded by a second detector. Neither dataset alone suffices to reconstruct an image; instead, the image is formed by analyzing correlations between the two^19^. A significant challenge hindering the application of ghost imaging to X-ray energies has been the generation of correlated photon pairs. Spontaneous parametric down-conversion (SPDC), the conventional method for producing entangled photons, suffers from extremely low conversion efficiency ($10^{-10}$–$10^{-11}$ per pump photon) and correspondingly low pair-generation rates at X-ray wavelengths^20–22^.

Positron annihilation offers a compelling alternative source of correlated photon pairs. When a positron annihilates with an electron, conservation of momentum dictates that two 511 keV photons are emitted in nearly opposite directions with precise temporal coincidence. This back-to-back emission geometry and intrinsic timing correlation have been exploited for decades in positron emission tomography (PET), where well-established coincidence detection infrastructure enables efficient identification of photon pairs^23^. Further, these annihilation photons are produced in an entangled Bell state with orthogonal polarization and correlated scattering kinematics^24,25^. While QAPI does not currently exploit these entanglement properties, the near-perfect momentum and temporal correlation of the photon pairs—arising from conservation laws governing the annihilation process—provides the physical basis for coincidence-based imaging. The additional quantum correlations remain available for future extensions, such as scatter discrimination via polarization-sensitive detection. Compared to SPDC, positron-emitting sources provide dramatically higher pair-generation rates: a modest 1 mCi clinical source produces approximately $10^{7}$ photon pairs per second with near-perfect angular and temporal correlation^26,27^, compared to only a few pairs per second achievable with SPDC at X-ray energies. Concurrent with the present work, Schneider et al.^28^ have experimentally demonstrated quantum ghost imaging using 511 keV photons from positronium decay, imaging high-density tantalum and tungsten objects with TOF-PET detector modules. Their results show that coincidence-based filtering substantially improves contrast and transmission accuracy over classical background subtraction, with the advantage most pronounced for low-transmission objects where background noise dominates. The present study complements this experimental demonstration by providing a rigorous statistical framework that identifies the fundamental mechanisms underlying the SNR advantage of coincidence-based imaging—namely, deterministic knowledge of the incident photon count and the application of binomial rather than Poisson transmission statistics—and validates these predictions through Monte Carlo simulation under both idealized and realistic detector conditions.

**Fig. 1:**
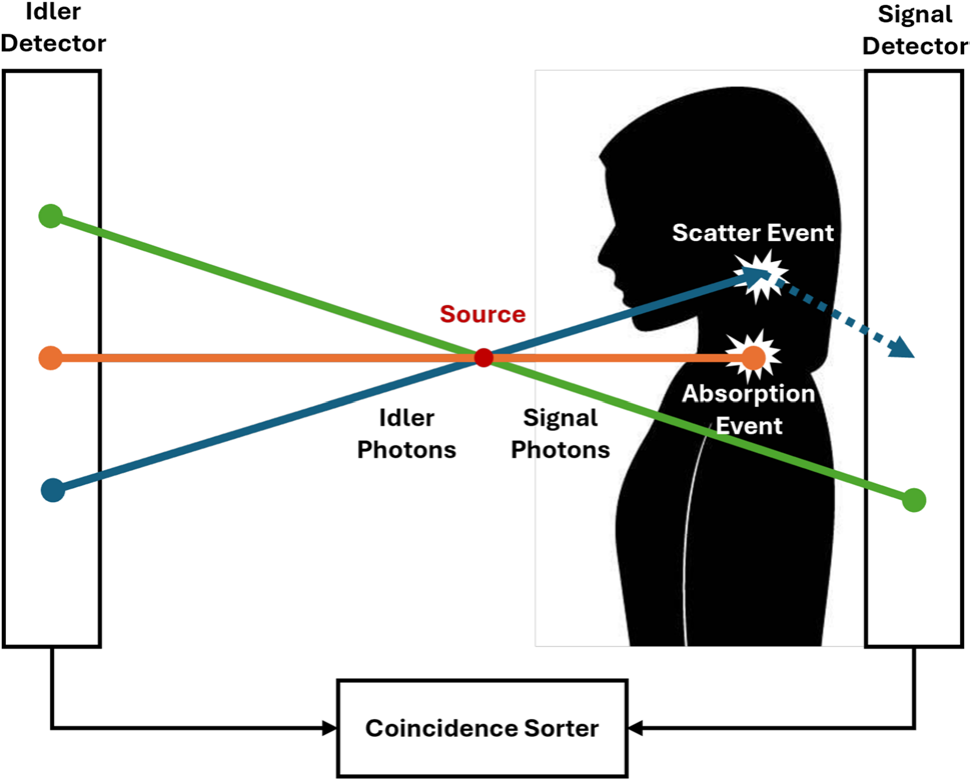
Illustration of the proposed Quantum Correlation of Annihilation Photons Projection. The diagram demonstrates the interaction pathways of annihilation photons emitted from a point source through an imaging object (e.g., chest or breast), resulting in transmitted, scattered, or absorbed photons. The photons detected by the coincidence sorter provide a high-resolution projection image using quantum correlations, enhancing diagnostic capabilities.

In this work, we propose Quantum-Assisted Photon Imaging (QAPI), an approach that integrates positron annihilation with ghost imaging principles to overcome the intrinsic noise limitations of classical transmission imaging. The QAPI geometry consists of a positron-emitting point source positioned between two opposing position-sensitive detectors, with the imaging object (e.g., breast or chest) interposed between the source and the signal detector. Following each annihilation event, one photon travels unimpeded to the idler detector while its correlated partner traverses the object before reaching the signal detector. For each detected idler event, the system records whether a coincident signal event is observed within a defined timing window. Events detected in coincidence indicate photon transmission through the object, while idler events lacking a coincident signal indicate absorption or scatter within the object, illustrated in fig. 1. The transmission image is then formed by computing, for each pixel, the ratio of coincident signal counts to total idler counts. This approach contrasts with classical transmission imaging, where the transmission image is computed by normalizing detected counts to an assumed source intensity. The key statistical advantage of QAPI arises from two factors. First, detection of idler photons provides an exact count of photons incident on the object, eliminating the uncertainty in photon generation that contributes to variance in classical imaging. Second, the high transmission probability of 511 keV photons permits application of binomial rather than Poisson transmission statistics, further reducing variance. Quantitatively, for a mean number of generated photons $\mu _g$ and transmission probability $p_t$, classical imaging yields a variance of $\mu _g p_t (1 + p_t)$, whereas QAPI achieves a variance of $\mu _g p_t (1 - p_t)$. The resulting SNR improvement grows as $p_t$ approaches unity, making QAPI particularly advantageous for high-transmission imaging scenarios such as breast or lung tissue. While these statistical benefits are substantial, we note that the dose implications of transitioning from conventional X-ray energies to 511 keV photons require separate investigation and remain an important consideration for clinical translation.

The following sections develop and validate the statistical framework underlying QAPI. We first derive analytical expressions for the photon-counting statistics of QAPI and classical imaging modalities, then validate these predictions through GATE Monte Carlo simulations under both idealized and realistic CZT detector conditions. The results establish a theoretical and computational foundation for quantum-assisted transmission imaging and motivate further experimental development toward practical implementation.

## Methods

### Statistics of photon counting in relation to QAPI

Consider a simplified photon counting experiment consisting of: (i) a pair of opposing ideal detectors (the Idler and the Signal detectors), (ii) a pencil beam photon source with a known average emission rate which emits back-to-back photons incident on the detector pair, and (iii) a material slab occluding the Signal detector. This minimal setup allows us to isolate and examine the fundamental statistics underlying photon counting in conventional radiation imaging modalities and compare against our proposal. We consider the cases of Classic (70 keV) and Classic (511 keV), where we do not have access to the Idler detector events, against that of QAPI (511 keV), where we do have Idler event information, noted as Models A, B, and C, respectively. The following derivations employ standard results from probability theory and counting statistics^29,30^.

The resulting statistical relationships are summarized in Table 1. In brief, we expect the intrinsic net variance of QAPI to be lower than that of classical tomography approaches thanks to differences in source and transmission statistics. Further, for a constant transmission probability, the mean number of detected photons remains unchanged across modalities. Consequently (same mean but lower variance), the signal-to-noise ratio (SNR) derived from the statistics of QAPI will always exceed that of a classical approach, irrespective of the photon generation rate.

It is important to note that these derived results intentionally omit secondary effects such as variations in intrinsic detection efficiency or detector technology. This simplification is by design: our goal here is to present a detector-agnostic analysis that isolates the statistical behavior of the imaging technique itself, independent of specific implementation details.

**Table 1:** Comparison of photon detection models, their statistical moments, and resulting SNRs.

Imaging model	Source model	Transmission model	Joint mean ($\mu$)	Joint variance ($\sigma ^2$)	SNR ($\mu / \sigma$)	Relative SNR vs. Model A
Model A	Poisson	Poisson	$p_t \mu _g$	$p_t \mu _g (1 + p_t)$	$\dfrac{\sqrt{p_t \mu _g}}{\sqrt{1 + p_t}}$	1
Model B	Poisson	Binomial	$p_t \mu _g$	$p_t \mu _g$	$\sqrt{p_t \mu _g}$	$\sqrt{1 + p_t}$
Model C	Known	Binomial	$p_t \mu _g$	$p_t \mu _g (1 - p_t)$	$\dfrac{\sqrt{p_t \mu _g}}{\sqrt{1 - p_t}}$	$\dfrac{\sqrt{1 + p_t}}{\sqrt{1 - p_t}}$

#### Transmission for a fixed number of photons

Using our simple experiment, first consider a case with a fixed number of emitted photons $n_g$. The number of transmitted photons $n_t$ follows a binomial distribution, as each photon has an independent probability $p_t$ of transmission, with conditional mean and variance:

1 $$\begin{aligned} n_t \,|\, n_g \sim \textrm{Binomial}(n_g, p_t), \quad \mathbb {E}[n_t \,|\, n_g] = n_g p_t, \quad \textrm{Var}(n_t \,|\, n_g) = n_g p_t (1 - p_t). \end{aligned}$$

In many imaging contexts, such as computed tomography, photon emission rates are high ($n_g \gg 1$) while the probability of transmission per photon is small ($p_t \ll 1$). In this regime, the binomial distribution approaches the Poisson limit. Formally, as $n_g \rightarrow \infty$ and $p_t \rightarrow 0$ such that the product $n_g p_t = \lambda _t$ remains constant, the binomial probability mass function converges to the Poisson form where $\lambda _t = n_g p_t$ represents the expected number of transmitted photons:

2 $$\begin{aligned} P(n_t = k \,|\, n_g) = \left( {\begin{array}{c}n_g\\ k\end{array}}\right) p_t^{k} (1 - p_t)^{n_g - k} \quad \rightarrow \quad P(n_t = k \,|\, n_g) = \frac{\lambda _t^k e^{-\lambda _t}}{k!}. \end{aligned}$$

So, the conditional distribution, mean, and variance become:

3 $$\begin{aligned} n_t \,|\, n_g \sim \textrm{Poisson}(n_g \, p_t), \quad \mathbb {E}[n_t \,|\, n_g] = n_g p_t, \quad \textrm{Var}(n_t \,|\, n_g) = n_g p_t. \end{aligned}$$

While the expected number of transmitted photons is identical between the binomial and Poisson descriptions, the variance differs by a factor $(1 - p_t)$. In the 511 keV photon cases (models B and C), the probability of transmission is great, precluding the Poisson model and benefiting from the tighter variance of the binomial model. This consequence is one of the core performance enhancers of QAPI. Further, this description assumes a known $n_g$. While QAPI benefits from an actual count of this value via the Idler detection events (the second intrinsic improvement), the classic techniques provide only estimates of this stochastic value. So, we must compute the joint distribution between source and transmission statistics to view the full picture.

#### Poissonian photon generation

Photon emission from a radioactive or quantum source is inherently stochastic and is well described by a Poisson process. The number of generated photons $n_g$ over a fixed acquisition period follows:

4 $$\begin{aligned} n_g \sim \textrm{Poisson}(\mu _g), \quad \mathbb {E}[n_g] = \mu _g, \quad \textrm{Var}(n_g) = \mu _g, \end{aligned}$$

where $\mu _g$ is the mean number of generated/emitted photons. In this thought experiment, we assume $\mu _g$ is known precisely. While this assumption is not strictly valid in all contexts, the emission rate in medical imaging systems is typically well characterized through calibration, and the uncertainty in $\mu _g$ is negligible compared to the intrinsic Poisson variability in the emissions. Here, QAPI provides an exact measure of $n_g$ while the classic techniques must use $\mu _g$ as a stand-in and contend with the fundamental variance inherent to that assumption.

#### Poissonian photon generation with Poissonian transmission

We now derive the joint expected mean and variance for a classical imaging case where both photon generation and transmission can be modeled as Poisson processes (Table 1 – Model A). Applying the previously defined moments described in Eqs. (3) and (4), we use the laws of total expectation and total variance to obtain:

5 $$\begin{aligned} \mathbb {E}[n_t]&= \mathbb {E}[\mathbb {E}[n_t \,|\, n_g]] = p_t \, \mathbb {E}[n_g] = \mu _g p_t, \end{aligned}$$

6 $$\begin{aligned} \textrm{Var}(n_t)&= \mathbb {E}[\textrm{Var}(n_t \,|\, n_g)] + \textrm{Var}(\mathbb {E}[n_t \,|\, n_g]) \end{aligned}$$

7 $$\begin{aligned}&= \mathbb {E}[n_g p_t] + \textrm{Var}(n_g p_t) \end{aligned}$$

8 $$\begin{aligned}&= p_t \mu _g + p_t^2 \mu _g = \mu _g p_t (1 + p_t). \end{aligned}$$

Thus, the expected number of detected photons is $\mu _g p_t$, and the variance is $\mu _g p_t (1 + p_t)$, reflecting the additional stochasticity introduced by compounding two Poisson processes.

#### Poissonian photon generation with binomial transmission

In the low $\mu _g$, high $p_t$ case, such as for low-count, high-energy studies (Table 1 – Model B) , we treat transmission as a binomial process rather than Poissonian. Applying the previously defined moments described in Eqs. (1) and (4), we use the laws of total expectation and total variance to obtain:

9 $$\begin{aligned} \mathbb {E}[n_t]&= \mathbb {E}[\mathbb {E}[n_t \,|\, n_g]] = p_t \, \mathbb {E}[n_g] = \mu _g p_t, \end{aligned}$$

10 $$\begin{aligned} \textrm{Var}(n_t)&= \mathbb {E}[\textrm{Var}(n_t \,|\, n_g)] + \textrm{Var}(\mathbb {E}[n_t \,|\, n_g]) \end{aligned}$$

11 $$\begin{aligned}&= \mathbb {E}[n_g p_t (1 - p_t)] + \textrm{Var}(n_g p_t) \end{aligned}$$

12 $$\begin{aligned}&= p_t (1 - p_t) \mu _g + p_t^2 \mu _g = \mu _g p_t. \end{aligned}$$

Thus, the expected number and variance of detected photons are both equal to $\mu _g p_t$. This result reflects the well-known property of Poisson thinning, wherein independent selection of events from a Poisson process with probability $p_t$ produces a new Poisson process with rate parameter $\mu _g p_t$.

#### Known photon generation with binomial transmission

In the final case, we assume that the number of generated photons $n_g$ is known (i.e., deterministic rather than stochastic), as in the QAPI case where we can count the number of emitted photons via the Idler detector (Table 1 – Model C). We additionally choose a binomial transmission process given the constraints of QAPI. Because $n_g$ is fixed, the conditional moments in Eq. (1) are also the unconditional moments. For comparison with the Poissonian generation cases, we may set $n_g = \mu _g$, yielding:

13 $$\begin{aligned} \mathbb {E}[n_t]&= n_g p_t = \mu _g p_t, \end{aligned}$$

14 $$\begin{aligned} \textrm{Var}(n_t)&= n_g p_t (1 - p_t) = \mu _g p_t (1 - p_t). \end{aligned}$$

Here, the variance is reduced relative to the Poissonian cases, as the deterministic generation process removes one source of stochasticity. The only randomness arises from the probabilistic transmission of each photon.

#### Summary of statistical findings

The preceding analysis reveals that while the mean number of detected photons remains constant across all three imaging models ($\mu _g p_t$), the joint variance is sensitive to the statistical regime in which one operates. Specifically, the choice of source and transmission models yields distinct variance expressions that differ by factors dependent on the transmission probability $p_t$.

For high-attenuation, high-flux imaging (Model A), the Poisson–Poisson formulation yields a variance inflated by a factor of $(1 + p_t)$ relative to standard Poisson statistics. In contrast, high-transmission scenarios capture binomial statistics (Model B) and recover the familiar Poisson-thinned variance. The QAPI paradigm (Model C) further reduces variance by a factor of $(1 - p_t)$ through elimination of source uncertainty, with the improvement becoming substantial as $p_t \rightarrow 1$.

These results are purely statistical in nature and intentionally abstracted from detector-specific effects. The analysis demonstrates that QAPI possesses an intrinsic SNR advantage arising from two compounding factors: (i) access to binomial rather than Poisson transmission statistics in the high-$p_t$ regime, and (ii) deterministic knowledge of the incident photon count. To validate whether these theoretical predictions manifest in realistic imaging scenarios, we now turn to Monte Carlo simulations that incorporate the full physics of photon transport.

### Idealized simulation methods

To validate the theoretical statistics presented above and quantify the expected improvement in SNR, we implemented a Monte Carlo simulation of an expanded scenario of the thought experiment above using GATE^31^. The simulation compares QAPI and classic transmission imaging under matched geometric and source conditions. In this experiment, our goal is not to reproduce realistic detector physics but rather to isolate the intrinsic statistical behavior of each imaging paradigm. The following section outlines a physically realistic scenario.

#### Simulation geometry and parameters

**Fig. 2:**
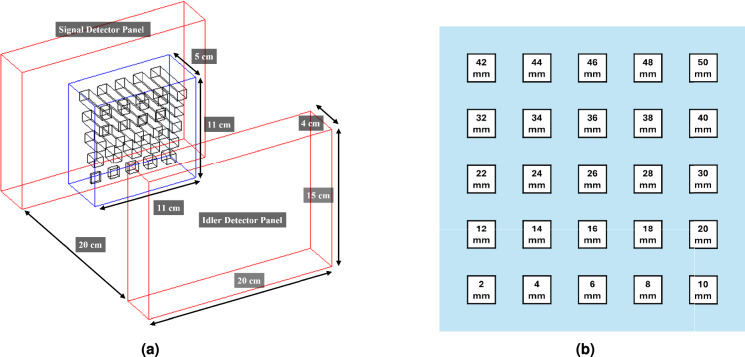
Visualization of the system and phantom in GATE (**a**). The signal panel sits behind the imaging target while the idler panel opposes it. The face geometry of the phantom with labeled thicknesses of each insert (**b**). (**a**) GATE geometry. (**b**) Phantom face geometry.

Two opposing detector panels, with face measurements of $20 \times 15~\mathrm {cm^2}$ and $1~\textrm{mm}$ pixel size, were placed $20~\textrm{cm}$ apart. Because GATE lacks a direct mechanism to enforce ideal detector behavior, we emulate perfect detection using a fictional material with a high atomic number ($Z=92$) and density ($\rho = 100~\mathrm {kg/cm^3}$). Time and energy blurring were disabled, and all detected events were grouped using ground-truth information to eliminate effects of coincidence ambiguity or unwanted noise. The GATE geometry is visualized in Fig. 2a.

The target object was a structured slab phantom designed to produce a range of transmission probabilities. The phantom volume ($11 \times 11 \times 5~\mathrm {cm^3}$) was composed of GATE material *body*, into which an array of lower-density inserts (GATE material *lung*) was embedded. Each insert measured $1 \times 1 \times d~\mathrm {cm^3}$ and was centered within the background material with $1~\textrm{cm}$ pitch. The insert depth *d* varied from 2 to $50~\textrm{mm}$ in $2~\textrm{mm}$ increments. Because the lung inserts are less dense than the surrounding body material, increasing insert thickness replaces more of the attenuating background with lower-density material, resulting in higher transmission probability. Thus, the thinnest inserts (2 mm lung, 48 mm body) correspond to the lowest transmission probabilities, while the thickest inserts (50 mm lung, 0 mm body) correspond to the highest. This design allows systematic evaluation of signal attenuation as a function of material thickness (probability of transmission). The GATE and face geometry of the phantom is visualized in Fig. 2.

An array of back-to-back pencil-beam $\gamma$ sources was positioned along the mid-plane between the opposing panels, such that each pixel pair had a corresponding source along the line joining their centers. Each source emitted photons along its respective pixel axis with an activity of $100~\textrm{kBq}$, corresponding to an average of 100 back-to-back emission pairs per ms. This source array is not visualized in Fig. 2aas it appears as a solid plane between the two detector faces. Two photon energies were simulated: $511~\textrm{keV}$ for the QAPI configuration and $70~\textrm{keV}$ for the classical transmission configuration. These energies were selected to reflect the operational regimes typical of annihilation-based and X-ray-based modalities, respectively.

#### Acquisition and data processing

For each imaging mode, a $100~\textrm{ms}$ acquisition was performed. Detected photon counts were tabulated into two matrices:

- **Idler matrix** (*I*): counts recorded in the unobstructed panel, representing the number of photons incident on each pixel pair. This count is equivalent to the number of photons generated, $n_g$.
- **Signal matrix** (*S*): counts in the obstructed panel after photon transmission through the phantom, corresponding to the number of transmitted photons, $n_t$.

Because event grouping relied on ground-truth information and the detectors were idealized, the only sources of randomness in the simulation were photon generation and photon transmission—directly mirroring the statistical framework derived earlier. Only full-energy deposition events were included, effectively rejecting scattered photons. Signal and Idler photons were grouped by their GATE *eventID*, which ties them to specified emission events, allowing perfect coincidence pairing and event sorting.

From these data, two imaging modes were reconstructed as a function of exposure time:

15 $$\begin{aligned} \text {QAPI transmission image:} \quad&T_\textrm{QAPI} = \frac{S}{I}, \end{aligned}$$

16 $$\begin{aligned} \text {Classical transmission image:} \quad&T_\textrm{classical} = \frac{S}{A \, t}, \end{aligned}$$

where *A* is the source activity and *t* is the exposure time. The former normalizes transmitted counts by the actual number of generated photons, while the latter assumes a constant source rate, as in traditional imaging. Here, we assume *A* is precisely known. The 511 keV data is used for both QAPI and classic imaging (independently) while the 70 keV data was used only in a classic capacity.

#### Performance metrics

We evaluated SNR as a function of both total photon count (analogous to exposure time) and insert depth (analogous to transmission probability). This comparison allows direct validation of the analytical expectations previously derived, demonstrating that the observed SNR improvement of QAPI arises naturally from its statistical structure rather than from detector-specific or source-specific effects.

### Realistic simulation methods

While the idealized simulation methods provide a focused examination of the pure statistical performance of QAPI in isolation from detector performance, physical imaging necessarily depends on detector characteristics, as the counting process itself contributes binomial variance. The magnitude of variance degradation depends upon the material properties of the detector and the supporting electronics. To capture these effects and demonstrate QAPI performance under realistic detector conditions, we perform an additional set of simulations with detector physics explicitly modeled.

#### Detector model

We model the two-panel system as comprising stacks of edge-oriented, $40\times 40\times 5~\mathrm {mm^3}$ Cadmium Zinc Telluride (CZT) crystals occupying the same panel volume as the idealized case. Each panel is composed of a $5\times 30$ array of these crystals, providing total active dimensions of $200\times 150~\mathrm {mm^2}$. GATE interaction events (hits) are recorded and processed with in-house software implementing event blurring, digitization, and coincidence sorting.

Temporal resolution is modeled by blurring hit times to 8 ns full width at half maximum (FWHM). Energy resolution is modeled as 5.85% FWHM at 511 keV, corresponding to typical CZT spectrometer performance^32^. Hits depositing less than 10 keV are discarded to simulate electronic noise thresholding. Hit interaction locations are discretized to 1 mm resolution to emulate cross-strip electrode architecture, wherein orthogonal electrode arrays on opposing crystal faces provide *x*- and *y*-coordinates from charge collection, while the charge ratio between faces yields the *z*-coordinate (depth of interaction)^32,33^.

Photons undergoing multiple interactions within the detector volume are grouped based on a 20 ns adder window. For grouped events, the interaction closest to the field of view (FOV) is taken as the primary interaction location, as timing resolution is insufficient to definitively identify the earliest interaction.

#### Simulation geometry and source configuration

**Fig. 3:**
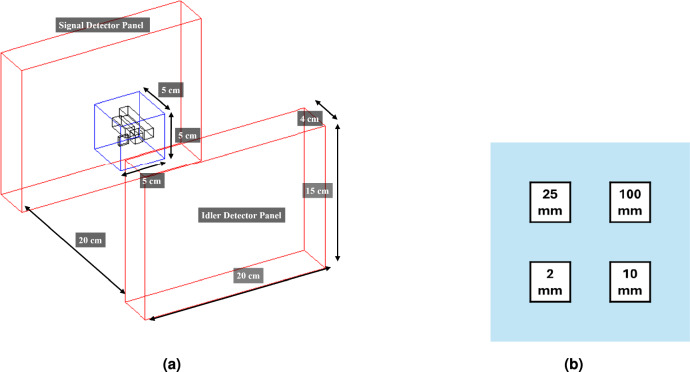
Visualization of the system and smaller phantom in GATE (**a**). The signal panel sits behind the imaging target while the idler panel opposes it. The face geometry of the reduced phantom with labeled thicknesses of each insert (**b**). (**a**) GATE geometry. (**b**) Phantom face geometry.

A 100 $\mu$Ci point source is positioned at the center of the FOV, emitting either back-to-back 511 keV annihilation photons or 70 keV photons depending on the imaging study. Because point-source acquisitions yield divergent projection images rather than the parallel-beam flat images of the idealized case, a smaller phantom is employed. The reduced phantom has a face area of $5\times 5~\mathrm {cm^2}$ with four inserts of depths 100, 25, 10, and 2 mm embedded in background material, as illustrated in Fig. 3. This geometry produces a range of transmission probabilities suitable for evaluating SNR performance.

#### Simulation studies

Five independent simulation studies were conducted to evaluate QAPI and classical imaging performance:

1. **QAPI (511 keV)**: Both signal and idler panels present, phantom in place, 5 min acquisition, energy window [470, 570] keV.
2. **Classic (511 keV)**: Signal panel only, phantom in place, 5 min acquisition, energy window [470, 570] keV.
3. **Air scan (511 keV)**: Signal panel only, no phantom, 10 min acquisition, energy window [470, 570] keV.
4. **Classic (70 keV)**: Signal panel only, phantom in place, 5 min acquisition, energy window [60, 80] keV.
5. **Air scan (70 keV)**: Signal panel only, no phantom, 10 min acquisition, energy window [60, 80] keV.

The air scan acquisitions provide reference normalization images for the classical reconstructions, analogous to blank scans in conventional transmission imaging. Extended acquisition times for air scans reduce statistical uncertainty in the normalization factors.

#### Image formation

Because photons penetrate some distance into the detector prior to detection, detected events must be back-projected along their incident paths to form coherent planar images. For each detected event, the vector pointing from the known source location (at the origin) to the detected interaction location defines the incident path. The surface of each panel is discretized into an image plane of $200\times 150$ pixels with $1\times 1~\mathrm {mm^2}$ pitch, and surface-projected hits are binned accordingly.

For classical imaging, no coincidence pairing is required; the pixel count matrices serve directly as the projection images. Transmission images are formed by dividing phantom acquisitions by the corresponding air scan images, pixel by pixel.

#### QAPI coincidence processing

The QAPI dataset requires coincidence pairing between signal and idler events. The following logic is applied:

- **Idler events**: All events in the idler detector are recorded, representing the total number of generated photons.
- **Signal events**: A signal event is counted for image formation only if a coincident idler event is detected within a 20 ns timing window. Signal events without a coincident idler (termed “lonely signals”) are flagged for potential future analysis but excluded from image reconstruction.
- **Geometric scatter rejection**: Because the trajectory of each photon pair is constrained by coincidence geometry, scatter events can be identified without relying on energy windowing. For each coincident pair, the expected signal trajectory is defined by the vector from the idler interaction location through the source to the signal plane. Signal events deviating significantly from this expected trajectory (>1 pixel width) are identified as phantom scatter and discarded. This geometric rejection enables operation without strict energy filtering, substantially improving detection sensitivity.
- **Low-energy event handling**: Low-energy idler events are assumed to represent partial energy depositions (e.g., Compton scatter within the detector) rather than scatter from the air gap, and are retained. Low-energy signal events may arise from either phantom scatter or partial energy deposition; the geometric criterion above distinguishes between these cases.

The QAPI transmission image is computed as the ratio of coincident signal counts to idler counts for each pixel pair, directly analogous to the idealized case but incorporating realistic detector response.

#### Performance metrics

For each imaging configuration, ROIs corresponding to each phantom insert and the surrounding background are defined using ground-truth geometry. Due to the expected projection artifacts in these images, the ROI covers a smaller area ($6\times 6~\mathrm {mm^2}$) than the actual insert area ($10\times 10~\mathrm {mm^2}$) to avoid such regions. This reduction accounts for the cone-beam projection geometry: because divergent photon paths near insert boundaries traverse mixed thicknesses of insert and background material, the projected edges exhibit transmission gradients that are not representative of the bulk insert attenuation. The central $6\times 6~\mathrm {mm^2}$ region ensures that sampled paths traverse approximately uniform material, providing a cleaner basis for SNR comparison across modalities. SNR is computed as described in the idealized methods, enabling direct comparison between modalities.

## Results

### Idealized simulation results

**Fig. 4:**
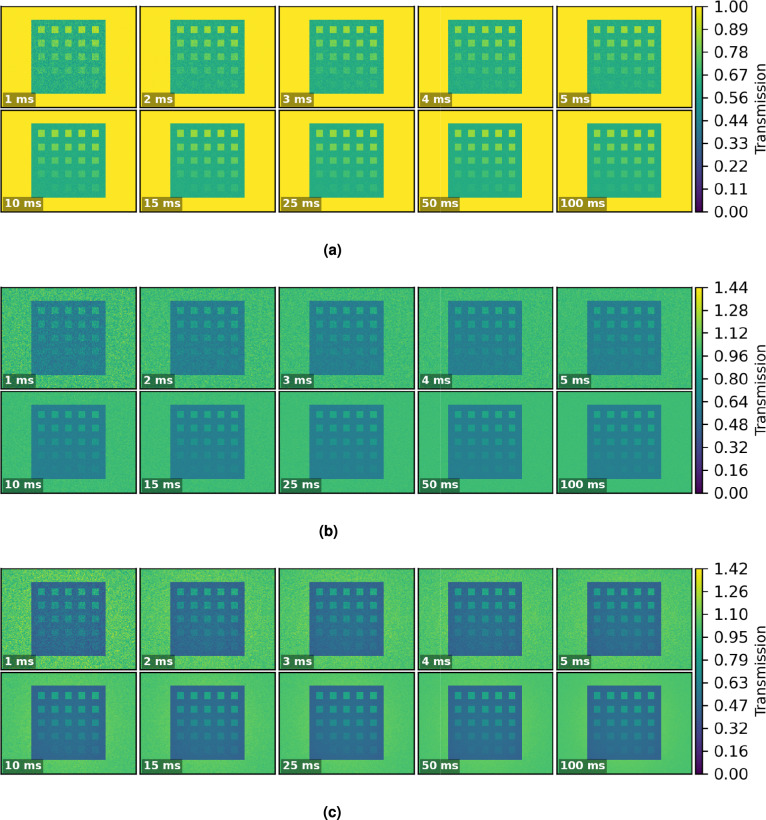
Transmission images for each imaging configuration. Each image corresponds to the same phantom and exposure conditions, demonstrating the dependence of observed contrast and noise characteristics on imaging modality and photon energy. (**a**) QAPI (511 keV). (**b**) Classic (511 keV). (**c**) Classic (70 keV).

Representative transmission images are shown for QAPI (511 keV) in Fig. 4a, classical (511 keV) in Fig. 4b, and classical (70 keV) in Fig. 4c, at exposure times spanning the 100 ms acquisition. As expected, photon energy strongly influences overall transmission. The mean background transmission was approximately 0.62 for the 511 keV cases and 0.49 for the 70 keV case, consistent with greater attenuation at lower energies. Transmission through each insert region followed the same trend, decreasing monotonically with insert thickness.

**Fig. 5:**
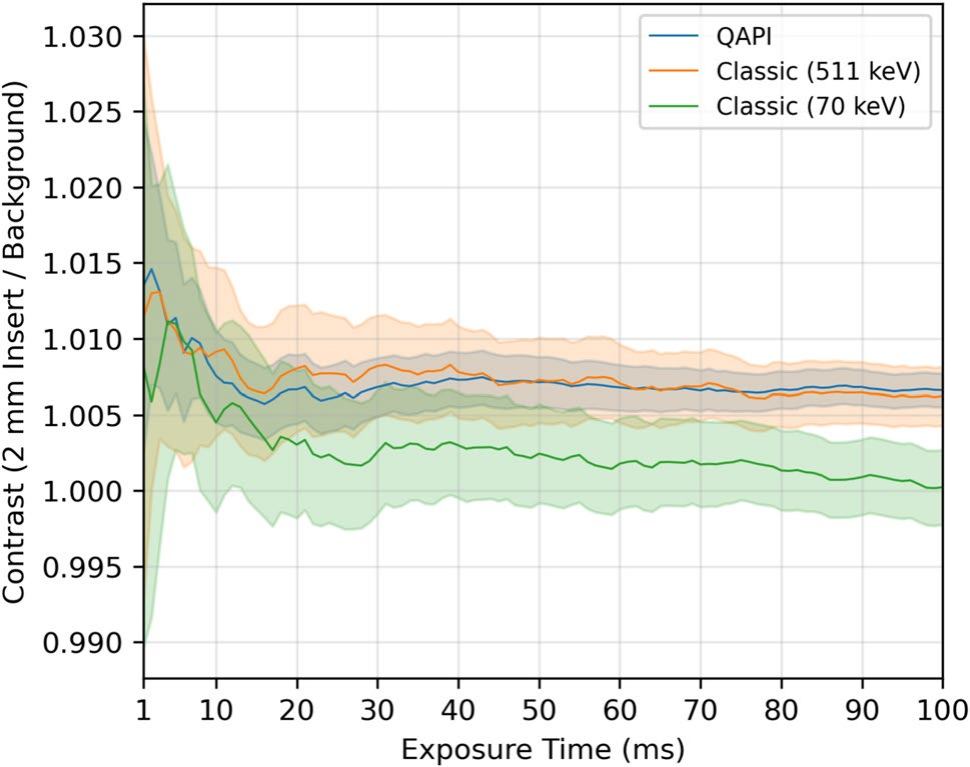
Contrast vs. exposure time for the smallest insert (2 mm lung, 48 mm body) for each imaging configuration.

Visually, contrast performance between the final images is comparable. The thinnest row of inserts (corresponding to the smallest transmission probabilities) is approximately as distinguishable across all three cases at the 100 ms mark (approximately 10,000 counts per pixel). Early frames favor the QAPI images, but this is attributable to noise performance rather than intrinsic differences in contrast. The transmission ranges for the classical approaches exceed unity, with peak pixel greater than 1.4 found in early frames (non-physical). Meanwhile, QAPI maintains a peak pixel value of exactly unity across the full exposure time. Quantitatively, QAPI achieves resolvable contrast against background for the smallest insert (2 mm lung, 48 mm body) at approximately $1.007\pm 0.001$ whereas the 70 keV classical imaging finds a contrast of $1.000\pm 0.0025$, as shown in Fig. 5.

**Fig. 6:**
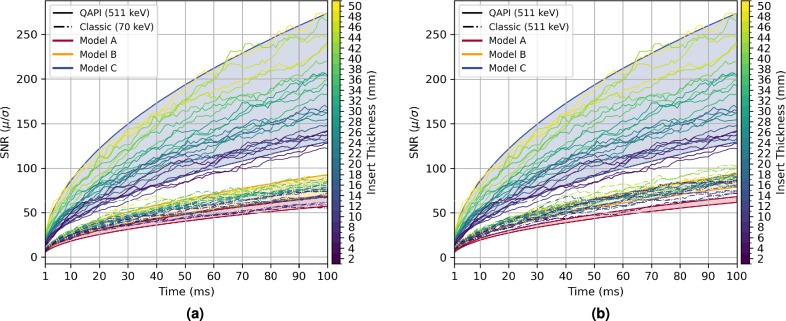
Idealized detector model. SNR of QAPI (511 keV) against Classic (70 keV) (**a**) and Classic (511 keV) (**b**) vs exposure time. The model ranges are defined by the most and least transparent lines and are defined as in Table 1. (**a**) QAPI (511 keV) against Classic (70 keV). (**b**) QAPI (511 keV) against Classic (511 keV).

For each insert in every imaging configuration, regions of interest (ROIs) were defined using ground-truth geometry, and the corresponding SNR was computed as a function of exposure time. The resulting trends are presented in Fig. 6b for QAPI (511 keV) against classical (70 keV) and in Fig. 6a for QAPI (511 keV) against classical (511 keV). Across all transmission probabilities and exposure durations, the measured SNR of QAPI consistently exceeds that of both classical imaging modes, in agreement with theoretical predictions. The shaded regions represent theoretical SNR bounds derived from the transmission probabilities of the most and least transparent inserts and using the SNR definitions presented in Table 1.

While we assigned Model A (Poisson source and Poisson transmission) to the 70 keV image in our Methods, the actual SNR performance better aligns with Model B (Poisson source and binomial transmission). The Poisson transmission model in A is fundamentally an approximation of the binomial model present in B, one which holds only under specific conditions. Here, the transmission probabilities of most inserts are great enough that the necessary assumption of a small $p_t$ fails, reverting the approximate Poisson model back to binomial. As such, these results are consistent with statistical expectations. Lower energy photons or a more attenuating target may better reveal this shift in statistics. Separately, the expected classic range in Fig. 6b appears to underestimate the thickest inserts and overestimate the thinnest. However, only seven of the 25 curves fall outside of this range—so, we attribute this observed behavior to random fluctuations rather than to model discrepancy.

**Fig. 7:**
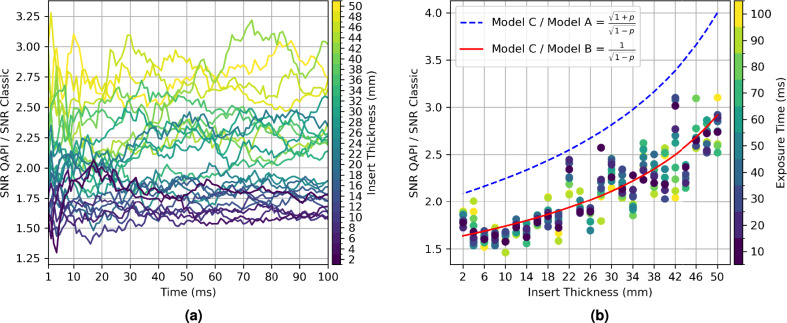
Comparison of the relative SNR of QAPI (at 511 keV) against Classic (at 511 keV) vs exposure time (**a**) and insert thickness (**b**). Here, exposure time is analogous to photon count while insert thickness is analogous to transmission probability. We expect the SNR ratio to be independent of time and dependent on insert thickness. The expected SNR ratio is given in red. (**a**) Relative SNR vs exposure time (photon count). (**b**) Relative SNR vs insert thickness (transmission probability).

Because the relative SNRs in Table 1 depend solely on transmission probability, the ratio of QAPI (511 keV) SNR to classical (511 keV) SNR, given by $1 / \sqrt{1 - p_t}$, should remain constant across exposure time. This behavior is confirmed in Fig. 7a: although the data are noisy, the SNR ratios are generally independent of time. Inserts with lower transmission probabilities exhibit lower SNR ratios, while those with higher transmission probabilities exhibit higher ratios, consistent with theoretical expectations. Plotting the same ratio as a function of insert thickness (analogous to transmission probability) in Fig. 7b reinforces this relationship, showing strong agreement between simulation and statistical predictions.

Because the transmission probabilities differ between the 511 keV and 70 keV trials, a direct SNR ratio comparison of QAPI (511 keV) and classic (70 keV) is not possible. Nevertheless, the observed SNR trends in all cases adhere closely to those predicted by the statistical framework, confirming that the expected model accurately describes the performance improvements afforded by QAPI.

### Realistic simulation results

Introducing realistic detector models adds fundamental degradations to system performance. Detector interactions follow a binomial process, increasing the expected variance and, more importantly, permitting full photon transmission through the detector volume. In classical imaging, detector transmissions are captured by the air scan and subsequently normalized out. In QAPI, however, these transmissions interfere substantially with coincidence pairing, leading to significant undercounting.

**Fig. 8:**
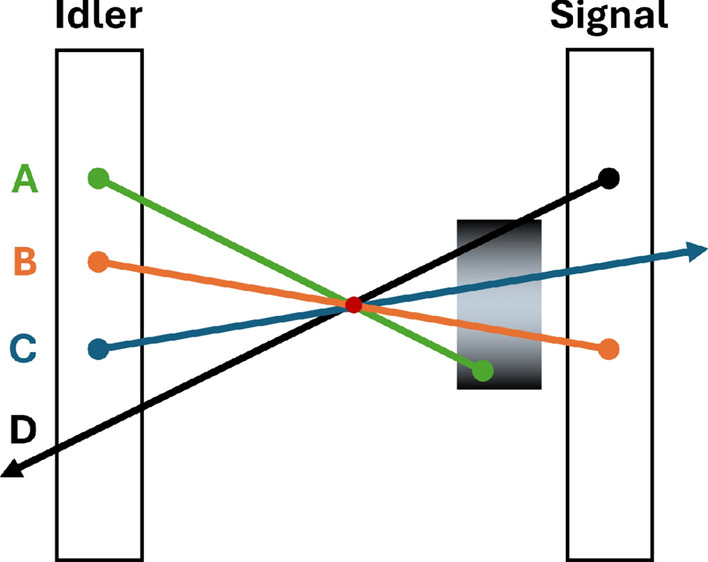
Possible scenarios for QAPI coincidences. Case **A** illustrates an idler-only event due to absorption or scatter within the phantom. Case **B** shows a coincidence event between the idler and signal detectors conveying actual transmission through the phantom. Cases **C** and **D** demonstrate transmission events through the signal and idler detectors, respectively, muddying the ability to compute noiseless transmission.

**Fig. 9:**
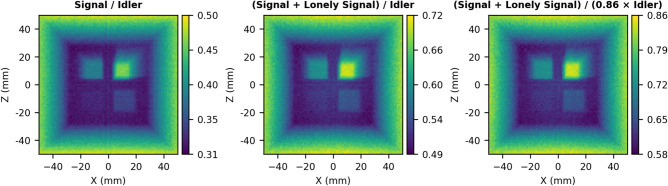
QAPI transmission image correction. (Left) The baseline signal/idler image substantially under-predicts transmission from detector transmit events. (Middle) By adding back in the energy gated signal-only events (the lonely signals), some contrast is recaptured. (Right) Correcting for the invisible signal losses by weighing the idler magnitude by its sensitivity to 511 keV photons, proper transmission is restored.

Figure 8 illustrates the possible scenarios for a single annihilation decay event. Cases **A** and **B** represent the expected system response: coincidence detection occurs (case **B**) unless the phantom attenuates the signal photon (case **A**). Beyond these desired outcomes, we must contend with events in which a photon is incident upon a detector but transmits through without interaction. Case **D** occurs when a signal event is observed with no corresponding idler event due to transmission through the idler detector—events we previously termed “lonely signals.” Such events cause undercounting of signal events and suppress the measured transmission ratio (the numerator in signal/idler decreases). Fortunately, after energy gating near 511 keV, we can infer where the corresponding idler photon should have been detected using the known system and source geometry, allowing us to artificially recover the missing idler count. Figure 9 demonstrates the transmission contrast recovered through this correction, zoomed in on the central $100\times 100~\mathrm {mm^2}$. Case **C**, however, cannot be corrected deterministically and is fundamentally indistinguishable from case **A**: signal photons that traverse the phantom but transmit through the signal detector without interaction appear identical to photons absorbed by the phantom. Case **C** undercounts signal events and further suppresses the measured transmission. We can partially compensate by weighting the idler matrix by the detector sensitivity to 511 keV photons (approximately 86%^34^) to rescale the transmission into the correct range, but this correction—unlike the lonely signal correction—does not remove the associated noise. Understanding and addressing this detector transmission problem is crucial for fully leveraging the statistical advantages of QAPI. The results presented hereafter incorporate both proposed corrections.

To characterize the performance of the geometric scatter rejection scheme, we evaluated accepted and rejected events against ground-truth interaction histories from the GATE simulation. Of all coincidence events, 84.2% were correctly accepted primaries, 6.4% were correctly rejected scatter events, 8.8% were primaries rejected by the geometric criteria, and 0.6% were scatter events incorrectly accepted, yielding a residual scatter fraction of 0.7% among accepted events. The geometric criterion thus retains 90.6% of true primaries while rejecting 91.3% of scatter-contaminated events. To assess the noise impact, we compared pixel-level standard deviations in the insert ROIs between the geometrically filtered images and ground-truth scatter-free images at full exposure time. The average standard deviation ratio (filtered to ground truth) across the four inserts was 1.041, indicating that the combined effect of residual scatter contamination and primary event losses contributes approximately 4% additional noise, a modest degradation that does not materially affect the SNR comparisons presented below. Different rejection criteria, such as altering the radial threshold or modifying the pixel size of the projected images, will affect the balance between scatter rejection and primary event retention, and consequently the net impact on SNR. Optimization of these parameters is an important direction for future work.

**Fig. 10:**
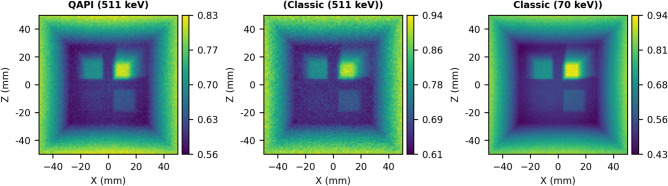
Transmission images from each scan type. The QAPI image uses the lonely signal and idler weighting corrections.

**Fig. 11:**
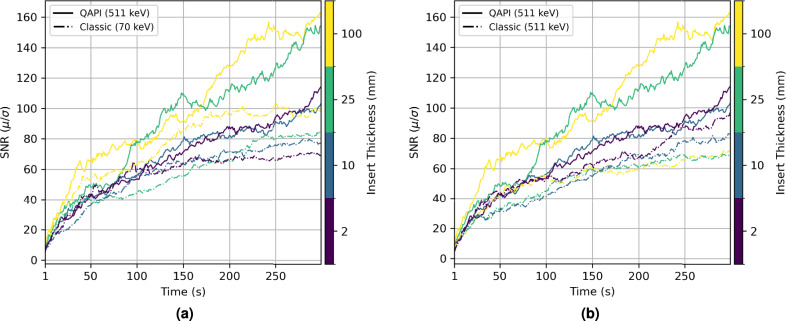
Realistic detector model. SNR of QAPI (511 keV) against Classic (70 keV). (**a**) and Classic (511 keV). (**b**) vs exposure time. (**a**) QAPI (511 keV) against Classic (70 keV). (**b**) QAPI (511 keV) against Classic (511 keV).

The 5-minute transmission images from each acquisition are shown in Fig. 10, zoomed in on the central $100\times 100~\mathrm {mm^2}$. Qualitatively, the 2 mm insert is most visible in the QAPI and 70 keV classical images and is nearly unresolvable in the classical 511 keV image. The noise texture of the QAPI image is visibly superior to that of the classical 511 keV image but appears inferior to that of the classical 70 keV image. However, the transmission range of the QAPI image (0.56 to 0.83) is narrower than that of the classical 511 keV (0.61 to 0.94) or classical 70 keV (0.43 to 0.94) images. This difference arises because the noisier processes in classical imaging produce higher variance transmission intensities and correspondingly larger maximum values; visually, the broader dynamic range can create an impression of smoother images. Quantitative SNR analysis confirms that the QAPI images achieve superior SNR performance relative to the classical images for all inserts (Fig. 11). QAPI improves SNR by a factor approaching $2\times$, and not far from the idealized improvement demonstrated in Fig. 7b.

## Discussion

This work presents a statistical framework and simulation-based validation of QAPI under both idealized and realistic detector conditions. By first isolating the underlying photon-counting statistics from detector- and system-specific effects, we demonstrate that the improved performance of QAPI arises intrinsically from its access to photon generation information via idler detector events. This formulation removes one source of stochasticity present in classical imaging, leading to a fundamental reduction in variance and an associated improvement in SNR.

Our idealized Monte Carlo simulations confirm the analytical predictions across a broad range of transmission probabilities and photon counts. In every configuration, QAPI exhibits superior contrast and SNR relative to classical imaging under equivalent photon flux and geometry. This advantage is particularly evident at short exposure times, where quantum noise dominates classical imaging performance. Importantly, the observed SNR ratios between QAPI and classical 511 keV modes match those predicted by the theoretical model, validating the derived relationship.

Small discrepancies between the predicted and simulated SNR ranges, particularly at the extremes of transmission probability, likely arise from model assumptions. For highly attenuating inserts (low $p_t$), stochastic variability in photon generation and finite sampling effects contribute to greater deviation from the expected Poisson–binomial limits. Conversely, for nearly transparent regions (high $p_t$), the simplifying assumption of independent transmission events breaks down as the Poisson model departs from its small-$p_t$ approximation. These deviations are minor and statistically consistent with the stochastic spread expected in finite Monte Carlo samples.

The realistic detector simulations reveal additional challenges that must be addressed for practical QAPI implementation. Chief among these is the problem of detector transmission: at 511 keV, approximately 14% of photons incident on a 4 cm CZT detector pass through without interaction. While classical imaging naturally accounts for such losses through air scan normalization, QAPI suffers from coincidence pairing failures when either the signal or idler photon transmits undetected. We identified two distinct failure modes: lonely signals (case **D**), where signal photons lack corresponding idler events, and undetected signals (case **C**), where signal photons traverse both the phantom and detector without interaction. The former can be corrected geometrically by inferring the missing idler location; the latter is fundamentally indistinguishable from phantom attenuation and introduces irreducible noise. Despite these complications, the realistic simulations demonstrate that QAPI retains a substantial SNR advantage—approaching $2\times$ improvement over classical imaging—after applying the proposed corrections. This result confirms that the theoretical statistical benefits of QAPI persist under realistic detector conditions, albeit with some degradation from the idealized case.

We additionally note that the recent experimental results of Schneider et al.^28^ provide independent support for the viability of coincidence-based imaging with annihilation photons. Their observed advantage is largest in the low-transmission regime, where background suppression via coincidence filtering is the dominant factor, while the statistical framework presented here predicts a growing intrinsic advantage at high transmission, where binomial variance reduction is most significant. Together, these findings suggest that coincidence-based annihilation photon imaging benefits from two complementary mechanisms: (1) background rejection and (2) intrinsic variance reduction, whose relative contributions depend on the transmission regime and detector noise environment. Notably, Schneider et al. identify imaging of very high transmission objects (>90%) as an important direction for future experimental work, precisely the regime in which the present framework predicts the largest intrinsic SNR advantage.

An important consideration not addressed in this work is the dose implications of transitioning from low-energy to high-energy photons. While 511 keV photons exhibit substantially higher transmission probabilities than conventional X-ray energies, they also deposit more energy per interaction, potentially increasing the delivered dose to the patient. Furthermore, the optimal source activity for QAPI must balance statistical power against random coincidence contamination, which scales quadratically with singles rate and becomes increasingly significant at high count rates. A rigorous dose-normalized SNR comparison between QAPI and conventional modalities requires careful treatment of the substantial differences in energy deposition characteristics, interaction cross-sections, and image formation—considerations that are beyond the scope of the present statistical foundations study but represent among the most important next steps for clinical translation of QAPI. Such analyses, along with count rate optimization and studies employing anatomically realistic phantoms, remain essential future work. While the statistical framework derived here depends only on the local transmission probability at each pixel and is therefore expected to generalize, heterogeneous anatomical structures may introduce additional scatter and spatially varying count rate effects that warrant dedicated investigation.

Together, the theoretical framework, idealized simulations, and realistic detector modeling presented here establish a strong foundation for quantum-assisted photon imaging. The consistent SNR improvements observed across all conditions validate the core principle that access to idler information provides a fundamental statistical advantage over classical transmission imaging. These results motivate continued development of QAPI toward clinical and preclinical applications where radiation dose reduction and improved low-contrast detectability are paramount. An important next step is experimental validation of the QAPI framework using a benchtop imaging system. Our group has previously developed and characterized cross-strip CZT detectors with the energy and timing resolution modeled in this work^32,35^, and adaptation of such a system to the dual-panel QAPI geometry is currently underway. A benchtop demonstration would allow direct verification of the predicted SNR improvements, evaluation of the proposed coincidence correction strategies under real detector noise conditions, and identification of any practical challenges—such as source collimation, background radiation, or count rate limitations—not captured by the current simulation framework. We anticipate that the statistical foundations and correction methods established here will directly inform the design and analysis of such experiments.

## Data Availability

The GATE simulation macros (with defined random seeds) and the GATE hit processing software is available at: https://github.com/RIL-Hub
